# Current Perspectives on Aerobic Exercise in People with Parkinson’s Disease

**DOI:** 10.1007/s13311-020-00904-8

**Published:** 2020-08-17

**Authors:** Sabine Schootemeijer, Nicolien M. van der Kolk, Bastiaan R. Bloem, Nienke M. de Vries

**Affiliations:** Donders Institute for Brain, Cognition and Behavior, Department of Neurology, Center of Expertise for Parkinson & Movement Disorders, Radboud University Medical Center, PO Box 9101, 6500 HB Nijmegen, Netherlands

**Keywords:** Exercise, Parkinson’s disease, endurance training, locomotion, health-related quality of life, mobility limitation

## Abstract

Parkinson’s disease (PD) is a progressive neurological disorder characterized by motor and non-motor symptoms for which only symptomatic treatments exist. Exercise is a widely studied complementary treatment option. Aerobic exercise, defined as continuous movement of the body’s large muscles in a rhythmic manner for a sustained period that increases caloric requirements and aims at maintaining or improving physical fitness, appears promising. We performed both a scoping review and a systematic review on the generic and disease-specific health benefits of aerobic exercise for people with PD. We support this by a meta-analysis on the effects on physical fitness (VO_2_max), motor symptoms (Movement Disorder Society Unified Parkinson’s Disease Rating Scale (MDS-UPDRS) motor section), and health-related quality of life (39-item Parkinson’s disease Questionnaire (PDQ-39)). Aerobic exercise has generic health benefits for people with PD, including a reduced incidence of cardiovascular disease, a lower mortality, and an improved bone health. Additionally, there is level 1 evidence that aerobic exercise improves physical fitness (VO_2_max) and attenuates motor symptoms (MDS-UPDRS motor section) in the off-medication state, although the long-term effects (beyond 6 months) remain unclear. Dosing the exercise matters: improvements appear to be greater after training at higher intensities compared with moderate intensities. We found insufficient evidence for a beneficial effect of aerobic exercise on health-related quality of life (PDQ-39) and conflicting results regarding non-motor symptoms. Compliance to exercise regimes is challenging for PD patients but may be improved by adding exergaming elements to the training program. Aerobic exercise seems a safe intervention for people with PD, although care must be taken to avoid falls in at-risk individuals. Further studies are needed to establish the long term of aerobic exercise, including a focus on non-motor symptoms and health-related quality of life.

**Electronic supplementary material:**

The online version of this article (10.1007/s13311-020-00904-8) contains supplementary material, which is available to authorized users.

## Introduction

Parkinson’s disease (PD) is a progressive neurological disorder characterized by both motor and non-motor symptoms. The well-known motor symptoms include bradykinesia, problems with gait and balance, tremor, and rigidity. The non-motor symptoms are diverse and can, among others, include fatigue, cognitive dysfunction, depression, and apathy. The prevalence of PD is vastly increasing, and by 2040, almost 13 million people worldwide will be affected by PD [1]. Unfortunately, there is neither a cure nor a disease-modifying treatment for PD. Current treatment mainly consists of dopaminergic replacement strategies (medication or deep brain surgery) aimed at alleviating PD symptoms. However, the effectiveness of the pharmacological treatment tends to wear off over time, resulting in disabling motor fluctuations and dyskinesias [2]. Moreover, not all patients are eligible for advanced therapies, which themselves are not without risks or side effects [3]. Taken together, there is a pressing need for alternative therapeutic strategies that reduce disability and improve health-related quality of life. Exercise (see Box 1 for a definition) is increasingly being recognized as an effective and highly promising non-pharmacological intervention to improve physical function. High-quality evidence on the disease-specific effects of exercise is relatively scarce [4], although several well-designed studies have been published in recent years.

**Box 1 Exercise terminology according to the American College of Sports Medicine**

**Table:** 

Term	Definition
Physical activity	Any bodily movement produced by the contraction of skeletal muscles that results in a substantial increase in caloric requirements over resting energy expenditure
Exercise	A type of physical activity that consists of planned, structured, and repetitive body movements that are performed to improve or maintain 1 or more components of physical fitness
Aerobic exercise	A subcategory of exercise that involves continuous movements of the body’s large muscles in a rhythmic manner for sustained periods

**Fig. 1:**
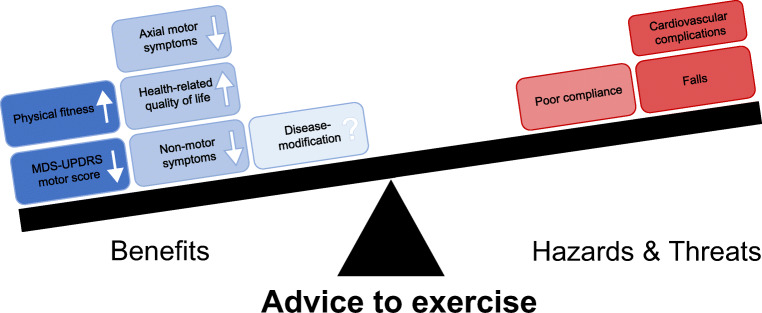
Benefits, potential hazards, and threats of aerobic exercise. The seesaw is tilting towards the left, indicating that the benefits of exercise outweigh the potential hazards and threats of aerobic exercise for people with PD. Darker blue color represents more evidence for benefit. The red color represents a potential hazard of exercise itself, while the lighter red color represents a threat to participating in exercise. There is a theoretical concern that elderly and sedentary persons who start a vigorous exercise program may experience cardiovascular complications, but this risk appeared to be very low in published exercise trials involving sedentary persons with PD. White arrows depict direction of change with aerobic exercise. MDS-UPDRS = Movement Disorders Society Unified Parkinson’s Disease Rating Scale.

In this review, we focus on aerobic exercise, a type of exercise that involves continuous movements of the body’s large muscles in a rhythmic manner for sustained periods, increases heart rate and caloric requirements, and is performed to maintain or improve physical fitness [5]. Different lines of evidence (animal models of PD [6–9], small human studies [10, 11], longitudinal cohort studies [12–16]) underlined the therapeutic potential of aerobic exercise in PD and identified aerobic exercise as a promising form of exercise to study in people with PD. A previous review of clinical trials investigating aerobic exercise in PD concluded that aerobic exercise improves physical fitness in PD, but evidence on disease-specific effects was lacking [17]. During the last years, the number of high-quality randomized clinical trials investigating the effect of aerobic exercise on PD symptoms (both motor and non-motor) has increased. In this comprehensive review, we provide an update of the current literature regarding the effect of aerobic exercise in people with PD (Fig. 1). In addition to the disease-specific effects of aerobic exercise, we also review the generic health benefits and issues related to implementation (safety and adherence).

## Methods

We review the effects of aerobic exercise on multiple domains in people with PD, including generic health benefits, disease-specific effects (on both motor and non-motor functioning), impact on health-related quality of life, and issues related to implementation. Because not all domains are studied in clinical trials, we combine narrative and systematic review techniques. The generic health benefits (cardiovascular health and risk factors, bone health, and fractures), prevention of PD, and implementation issues (safety and adherence) are described in a narrative way. The effects of aerobic exercise on physical fitness, on disease-specific motor and non-motor functioning, and on health-related quality of life are addressed in a systematic review. For the latter, we built upon a review [17] that included aerobic exercise trials published between 1990 and August 1, 2014. We systematically searched for randomized clinical trials from August 1, 2014 (i.e., the date of the search of the previous review [17] until February 19, 2020. The search strategy is available in Supplementary Box 1. In addition, we performed citation tracking through the included randomized clinical trials. We used similar inclusion criteria as before [17]: 1) target population consisting of people with PD, 2) aerobic exercise group exercising with at least an intensity of 60% of maximal heart rate (HRmax) [18], 3) intervention lasting at least 4 weeks, and 4) an aerobic exercise group was compared with a control or other non-aerobic exercise groups. We excluded one study [19] that was included in the earlier review [17] because of a lack of control or other non-aerobic exercise groups.

To further support the evidence, we conducted a meta-analysis for a few selected outcomes: physical fitness (maximal oxygen consumption, VO_2_max), motor function (Movement Disorder Society Unified Parkinson’s Disease Rating Scale (MDS-UPDRS) motor section) in the on- and off-medication state and health-related quality of life (39-item Parkinson’s Disease Questionnaire (PDQ-39)). We used the post-intervention scores of the exercise group and control group (between-group difference) to calculate a pooled mean difference (MD) with 95% confidence intervals (CIs) of the VO_2_max and PDQ-39. For the (MDS-)UPDRS motor section, we calculated the standardized mean difference (SMD) scores, because we had to combine two different outcome measures (the UPDRS motor section and the MDS-UPDRS motor section). Post-intervention scores were used, because they were presented in most of the studies and change scores were often not available. (Standardized) mean differences were pooled with the inverse variance method. Homogeneity of variances was evaluated with the *I*^2^ test [20], and if heterogeneity was absent (*I*^2^, 0–25%), we applied a fixed effects model. Heterogeneity was present for the (MDS-)UPDRS motor section; for this reason, we applied a random effects model. We examined funnel plots to assess the presence of publication bias, but the risk of publication bias was low [21].

**Fig. 2:**
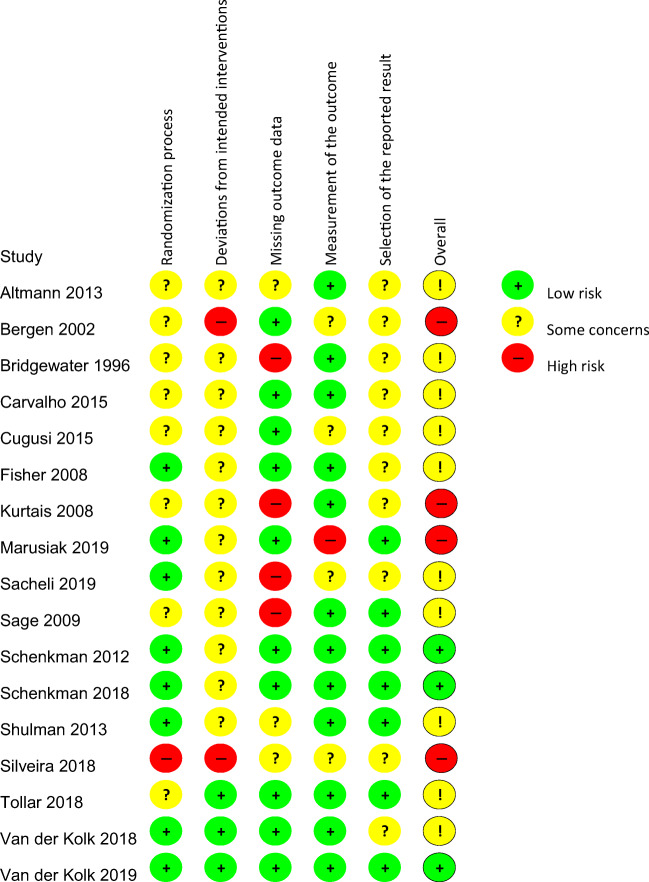
Summary of the risk of bias of randomized clinical trials included in the systematic review.

### Quality of Included Studies

The quality of the included randomized clinical trials was assessed by two reviewers (SS and NMdV) independently with the risk of bias tool [22]. Quality assessments were compared and discussed when necessary. In total, three studies were rated with the low risk of bias [4, 23, 24], 10 studies with some concerns about the risk of bias [11, 25–33], and four studies with the high risk of bias [34–37] (Fig. 2). The risk of bias was mainly based on 1) unclear randomization procedure and concealment; 2) lack of blinding of participants, therapists delivering the intervention and assessors; 3) unclear or incomplete reporting of dropouts; and 4) lack of a published prespecified statistical analysis plan. Blinding participants and therapists is often challenging when delivering an aerobic exercise intervention. Very few trials included a non-exercise group that received similar social interaction to blind the participants. In general, the newer studies were of higher quality than the older studies.

## Results

Table 1 provides an overview of the methods and major findings of the 17 randomized clinical trials included in this systematic review. Figure 1 provides an overview of the overall evidence for the effect of aerobic exercise. We suggest that the benefits of aerobic exercise outweigh the potential hazards and risks.

**Table 1:** Overview of studies on the effect of aerobic exercise in people with PD

Study	Sample	Intervention	Primary outcome	Tested on/off	Summary of results	Weaknesses	Risk of bias
Altmann (2016)	Total, 30 HY, 1–3 EX, 11 Active control (CON), 9 CON, 10	3 arms, 16 weeks EX: treadmill walking 3 times/week, 45 min/session, 50–75% HRR, personal supervision Active-CON: stretching and balance exercises 3 times/week, 45 min/session, personal supervision CON: usual care	Reaction time at speed of processing task and accuracy at accuracy/memory task	On	EX significantly improved in executive function in single task, and CON remained stable. No change in EX in dual task; CON deteriorated EX and active-CON remained stable in depressive symptoms, CON deteriorated	Small sample size, no intention-to-treat analysis, sample highly educated, lack of blinding of the control group	Some concerns
Bergen (2002)	Total, 8 HY, 2 EX, 4 CON, 4	3 arms, 16 weeks EX: cycle ergometry and treadmill walking 3 times/week, 40 min/session, 60–70% HRmax, supervised CON: normal activity level	Peak VO_2_ and movement initiation time	On	EX significantly improved VO_2_peak and movement initiation time compared to CON	Small sample, differences between EX and CON at baseline, CON received no alternative intervention (so less attention than EX), randomization allocation not specified	High risk
Bridgewater (1996)	Total, 26 HY, 1–3 EX, 13 CON, 13	2 arms, 16 weeks EX: walking exercises 2 times/week, 30 min/session, 65–85% HRmax, supervised CON: interest talks every 3 weeks, normal activity level	Not specified	Not specified	No change in motor symptoms (WRS) EX significantly improved stress test duration, NUDS, and HAP compared to CON No change in WRS	Small sample, differences between EX and CON at baseline, randomization allocation not specified, primary outcome not specified, medication state during assessments not specified	Some concerns
Carvalho (2015)	Total, 22 HY, 1–3 EX, 5 Strength, 8 CON, 9	3 arms, 12 weeks EX: treadmill walking 2 times/week, 30 min/session, 60–70% HRmax, supervision Strength: strength training 2 times/week, 30 min/session, 70–80% 1-RM, supervision CON: calisthenics 2 times/week, 30–40 min/session	UPDRS III	Not specified	EX and strength significantly improved in functional capacity (2MST), motor symptoms (UPDRS III), and increased cortical activity, no change in CON	Small sample, short intervention, no information on randomization, medication state not specified	Some concerns
Cugusi (2015)	Total, 20 HY, 1–3 EX, 10 CON, 10	2 arms, 12 weeks EX: Nordic walking 2 times/week, 60 min/session, 60–80% HRR, supervised group sessions CON: usual care	Not specified	On	EX significant improved in general fitness, motor function (improved strength, faster TUG, improvement balance), and non-motor symptoms (less depressive symptoms, apathy and fatigue) compared to CON	Small sample, short intervention Lack of blinding of the control group	Some concerns
Fisher (2008)	Total, 30 HY, 1–2 EX-high intensity, 10 EX-low intensity, 10 CON, 10	3 arms, 8 weeks EX-high intensity: body weight supported treadmill walking 3 times/week, 45 min/session, 75% HRmax, supervised EX-low intensity: conventional physical therapy 3 times/week, 45 min/session, 50% HRmax, supervised CON: 6 education classes	Not specified	Not specified	No between-group differences tested Motor symptoms (UPDRS III) decreased in all groups. Gait (speed, stride length) improved in EX-high intensity (HI); maximal cortical silent period increased in EX-HI	Small sample, short intervention, primary outcome not specified, medication state during assessments not specified, no between-group analysis	Some concerns
Kurtais (2008)	Total, 30 (24 analyzed) HY, 1–3 EX, 12 CON, 12	2 arms, 6 weeks EX: treadmill training 3 times/week, 40 min/session, 70–80% HRmax, supervised CON: not specified	Not specified	On	No between-group differences tested EX significantly improved at functional lower-extremity tasks and physical fitness (VO_2_peak). More people in EX rated their global physical status as better compared to CON	Small sample, short intervention, primary outcome not specified, no between-group analysis, no intention-to-treat analysis, treatment CON not specified	High risk
Marusiak (2019)	Total, 20 mHYS, 1.5–3 EX, 10 CON, 10	2 arms, 8 weeks EX: cycle ergometry 3 times/week, 60 min/session, 60–75% HRmax, supervised CON: conventional physiotherapy	Not specified	Off	EX improved psychomotor behaviors (bimanual motor control, executive function, neurological parkinsonian signs) compared to CON	Small sample, short intervention, assessor bimanual control not blinded	High risk
Sacheli (2019)	Total, 35 HY, 1–3 EX, 20 CON, 15	2 arms, 3 months (36 sessions) EX: cycle ergometry 3 times/week, 40–60 min/session, 60–80% VO_2_max, supervised CON: stretching 3 times/week, 40–60 min/session, supervised	Not specified	On and off	EX significantly improved physical fitness (VO_2_max) compared to CON. EX altered the responsivity of the ventral striatum, increased evoked dopamine release in the caudate nucleus	Small sample, short intervention, blinding assessor clinical measures not specified	Some concerns
Sage (2009)	Total, 46 UPDRS III, < 35 EX, 13 EX-sensory, 18 CON, 15	3 arms, 12 weeks EX: semi-recumbent elliptical exercise 3 times/week, 30 min/session, 60–75% HRmax, supervised EX-sensory: PD SAFEx program (non-aerobic gait and sensory attention exercises) 3 times/week, 40–60 min/session, supervised CON: waitlist	UPDRS III, TUG, spatiotemporal variables of self-paced gait	On	EX significantly improved gait (step length) compared to CON	Small sample, substantial dropout	Some concerns
Schenkman (2012)	Total, 121 HY, 1–3 EX, 41 EX-flexibility, 39 EX-home, 41	3 arms, 16 months EX: treadmill, cycle ergometry or elliptical trainer, 5–7 times/week, 30 min, partly supervised EX-flexibility: flexibility, balance, and functional exercise, 5–7 times/week, partly supervised EX-home: exercise at home using the National Parkinson Foundation Fitness Counts program, 5–7 times/week, 1 time/month supervision	Overall physical function (CS-PFP), balance (Functional Reach Test), walking economy (oxygen uptake)	On	EX-flexibility significantly improved in CS-PFP compared to EX after 4 months. No changes in balance EX significantly improved walking economy compared to EX-flexibility after 4 months, 10 months, and 16 months	No non-exercise control group, multiple primary outcome measures specified	Low risk
Schenkman (2018)	Total, 128 HY, 1–2, within 5 years of diagnosis EX-HI, 43 EX-moderate intensity (MI), 45 CON, 40	3 arms, 24 weeks HI: treadmill walking 4 times/week, 30 min/session, 80–85% HRmax, partly supervised MI: treadmill walking 4 times/week, 30 min/session, 60–65% HRmax, partly supervised CON: waitlist, usual care	Adherence to prescribed HR and frequency, safety, mean change in UPDRS motor score	Off	Good adherence to target HR and frequency Safe program EX-HI significant less deterioration motor symptoms (MDS-UPDRS III) compared to CON	Patients with only recent diagnosis and HY 1–2	Low risk
Shulman (2013)	Total, 67 HY, 1–3 HI, 23 MI, 22 Strength, 22	3 arms, 3 months HI: treadmill walking 3 times/week, 30 min/session, 70–80% HRR, supervised MI: treadmill walking 3 times/week, 50 min/session, 40–50% HRR, supervised Strength: resistance machines 3 times/week, 2 sets of 10 reps/leg, supervised	6MWT, VO_2_max, muscle strength (1-repetition maximum strength)	On	3 groups show similar improvements on 6MWT. HI and MI significantly improved physical fitness (VO_2_peak) compared to strength. Strength significantly improved muscle strength compared to HI and MI	No non-exercise control group, duration sessions HI, and MI differed	Some concerns
Silveira (2018)	Total, 76 HY, not specified EX, 29 Goal-based, 28 CON, 19	3 arms, 12 weeks EX: 3 times/week, 1 h/session, intensity started at 40–50% HRR, increased to 60–70% HRR, cycle ergometry, supervised Goal-based: 3 times/week, 1 h/session, PD SAFEx program (walking, muscle toning, whole body stretching) CON: usual care	All outcomes of neuropsychological tests in 5 cognitive domains	On	EX had significantly better inhibitory control (Stroop Test) at post-test in cognitively normal and impaired individuals compared to CON. EX had significantly better performance at post-test TMTB compared to CON in the cognitive impaired compared subgroup. PD patients with and without cognitive impairment benefit from aerobic exercise	Small sample and large variability in the cognitive impaired group, assessor VO_2_max not blinded, participants not blinded to group allocation when recruited, large dropout after randomization	High risk
Tollar (2019)	Total, 74 HY, 2–3 EX, 25 Agility, 25 CON, 24	3 arms, 5 weeks EX: cycle ergometry 5 times/week, 1 h/session, 80% HRmax, supervised group sessions Agility: Xbox games to improve postural control, gait mobility, gait stability, turning, and dynamic and static balance 5 times/week, 80% HRmax, supervised groups sessions CON: waitlist	MDS-UPDRS II	On	EX and agility significantly improved motor experiences of daily living (MDS-UPDRS II), health-related quality of life (PDQ-39), and depressive symptoms (BDI) compared to controls. Improvement MDS-UPDRS II exceeded minimal clinically important difference	Short intervention, physical activity not assessed, but patients were physically untrained, so therefore, there was possibly a similar effect of exercise programs. Possible placebo effect due to a lack of blinding of the control group	Some concerns
Van der Kolk (2018)	Total, 37 HY, 1–2 EX, 22 CON, 15	2 arms, 24 weeks EX: cycle ergometry 3 times/week, 45 min/session at 60–80% HRR, remote supervision CON: usual care	Adherence and adverse events	Off (VO_2_max on)	Adherence excellent, intervention feasible and safe	Small sample, effectiveness not tested	Some concerns
Van der Kolk (2019)	Total, 130 HY, 1–2 EX, 65 CON, 65	2 arms, 24 weeks EX: cycle ergometry 3 times/week, 30–45 min/session, 50–80% HRR, remote supervision CON: stretching exercises 3 times/week, 30 min/session, remote supervision	MDS-UPDRS III	Off (VO_2_max on)	EX significantly attenuated motor symptoms compared to CON (MDS-UPDRS III) and significantly increased physical fitness (VO_2_max) compared to CON	Patients with only HY 1–2 Improvements in MDS-UPDRS III did not transfer to on state	Low risk

BDI = Beck Depression Index; CON = control group; CS-PFP = Continuous Scale-Physical Functional Performance; EX = aerobic exercise group; HAP = human activity profile; HRmax = maximum heart rate; HRR = heart rate reserve; HY = Hoehn and Yahr Scale; MDS-UPDRS III = Movement Disorders Society Unified Parkinson’s Disease Rating Scale motor section; NUDS = Northwestern University Disability Scale; PDQ-39 = 39-item Parkinson’s Disease Questionnaire; TUG = Timed Up and Go; UPDRS III = Rating Unified Parkinson’s Disease Scale motor section; VO_2_max = maximum oxygen uptake; WRS = Webster Rating Scale; 1-RM = one-repetition maximum; 2MST = 2-min step test; 6MWT = 6-min walk test

### Generic Health Benefits

#### Cardiovascular Health

Aerobic exercise has generic health benefits for almost everyone, including people with a chronic disease like PD [5]. In older adults, regular physical activity is associated with a lower risk of cardiovascular disease and cardiovascular mortality [5]. There is a clear dose–effect relation between aerobic exercise and cardiovascular health and mortality: the more time spent on aerobic exercise at moderate intensity, the lower the cardiovascular mortality [38]. For exercise at vigorous intensity, the same dose–effect relationship holds true, except beyond 11 MET h/week, which does not lead to a further reduction in cardiovascular mortality [38].

Aerobic exercise also impacts on the metabolic system. Metabolic syndrome is characterized by the occurrence of several risk factors for cardiovascular disease (insulin resistance, hyperlipidemia, insulin resistance, hypertension) [39]. Exercise can prevent metabolic syndrome and can also result in reversal of muscle insulin resistance and reduction of postprandial lipogenesis [39]. These findings are also relevant for PD. Specifically, in one cohort study (*N* = 1.022), metabolic syndrome was associated with a faster deterioration in motor symptoms of PD [40]. Another cohort study found an association with cognitive decline in PD patients [41]. However, whether exercise can revert the metabolic syndrome (and its consequences) in PD patients remains to be studied.

Another interesting area relates to cerebral small vessel disease, including lacunar infarcts and white matter hyperintensities, which commonly occur as comorbidity in PD patients [42, 43]. Importantly, the concurrent presence of small vessel disease is associated with a poorer course of PD, with worsening of gait problems, cognitive decline, and depression [42, 44]. A lack of physical activity among PD patients may further aggravate this risk of developing comorbid small vessel disease. Theoretically, exercise could mitigate this risk, certainly when extrapolating the observed protective effects in the general population. However, a prophylactic effect of aerobic exercise on cerebrovascular disease has thus far not been studied in persons with PD.

#### Bone Health and Fractures

Regular physical activity is associated with improved bone health in older adults [5]. People with PD are generally less physically active than their healthy counterparts, which may contribute to the development of osteoporosis in PD [45]. Indeed, osteoporosis is very common in people with PD [46], who have a lower bone mineral density and reduced bone strength compared with healthy people [45, 47]. Apart from inactivity, other contributing factors include vitamin D deficiency, reduced muscle strength, poor mobility, and hyperhomocysteinemia (i.e., catabolism of homocysteine, a status associated with risk of fractures and a low bone mineral density; this status is possibly aggravated by levodopa use) [45]. Along with their gait deficits and postural instability (leading to falls), osteoporosis places people with PD at risk of sustaining fractures [46]. Moreover, the combination of PD and osteoporosis has been associated with pain, sleep problems, depression, and anxiety [48]. These consequences might be countered by weight-bearing aerobic exercise (brisk walking, running), which stresses the bones and thereby promotes bone growth and bone strength in healthy older adults [5]. The effect of aerobic exercise on bone health and osteoporosis in PD remains to be shown, but simple interventions such as brisk walking seem promising.

### Preventing Parkinson’s Disease

Epidemiological studies suggest that in the general population, people who are physically more active may be at lower risk of developing PD [13–16, 49–52]. Interestingly, premorbid physical activity also seems to influence disease symptom onset [52, 53]. For example, the chance of developing prodromal features of PD (constipation, bodily pain, depression, and excessive daytime sleepiness) is negatively associated with physical activity [54], as is motor symptom onset [53]. It is unclear whether physical activity and aerobic exercise can actually prevent or delay the development of PD itself (i.e., by slowing the underlying neurodegenerative process itself) or whether this induces compensatory effects in other brain areas that delay the manifestation of PD symptoms. Further research into the underlying mechanisms of the potential neuroprotective effect of physical activity and exercise is needed [55] and is also elaborated upon in the discussion of this review.

### Symptomatic Effects in Manifest Parkinson’s Disease

#### Physical Fitness

**Fig. 3:**
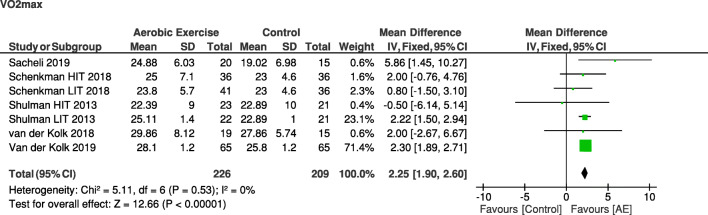
Meta-analysis on the effect of aerobic exercise on physical fitness in PD comparing post-intervention VO_2_max between the aerobic exercise group and the control group. AE = aerobic exercise group; HIT = high-intensity group; LIT = low-intensity group.

Aerobic exercise improves physical fitness in people with PD (Fig. 3). Ten randomized controlled trials investigated the effect of aerobic exercise on physical fitness, as assessed with a (sub)maximal graded exercise test (VO_2_max) [4, 11, 23, 24, 29, 31, 33–35, 37]. All studies showed a beneficial effect of aerobic exercise on physical fitness, although two studies only reported within-group effects [11, 35]. Different exercise types were studied but generally involved (treadmill) walking or cycling on a stationary bicycle. The studies also differed in length, varying from 6 to 24 weeks, and used different training frequencies (3–7 times/week) and intensities (60–85% HRmax, 60–80% VO_2_max, or 50–80% heart rate reserve). Two studies investigated the difference in effectiveness of low/moderate-intensity *versus* high-intensity aerobic exercise training [23, 31]. One of these studies showed similar improvements in physical fitness. However, in this study, the duration of the training sessions also differed, which makes it impossible to conclude whether duration or intensity of the exercise contributed to these effects [31]. The other high-quality trial showed that high-intensity exercise for 6 months was more effective in improving physical fitness than moderate-intensity exercise [23]. Overall, these studies provide level 1 evidence for a beneficial effect of aerobic exercise on physical fitness in PD. This positive effect is also supported by our meta-analysis (Box 2).

**Box 2 Results of meta-analyses**

**Table:** 

Physical fitness	The positive effect of aerobic exercise on physical fitness is supported by the meta-analysis: the aerobic exercise group showed a higher physical fitness during a maximal graded exercise test (VO_2_max) at the post-assessment compared with a non-exercise or resistance exercise control group (mean difference (MD) [95% CI], 2.25 [1.90; 2.60]; *Z* = 12.66; *p* < 0.05) (Fig. 3)
Motor symptoms in PD	The meta-analysis on (MDS)-UPDRS shows that patients with PD who have performed aerobic exercise experienced less motor symptoms at the post-assessment compared with the a non-aerobic control group when assessed in the *off* state [4, 23, 29, 33] (SMD [95% CI], − 0.42 [− 0.77; − 0.08]; *Z* = 2.39; *p* = 0.02) (Fig. 4B), but not when assessed in the *on* state [24, 25, 28, 30, 31] (SMD [95% CI], − 0.21 [− 0.72; 0.30]; *Z* = 0.81, non-significant) (Fig. 4A). Two studies [11, 27] were excluded from the analysis of the (MDS-UPDRS) because medication state was not reported
Health-related quality of life	People in the aerobic exercise group did not experience a better health-related quality of life at the post-assessment compared with those in the non-aerobic control group (MD [95% CI], − 0.31 [− 1.05; 0.43]; *Z* = 0.82, non-significant) (Fig. 5)

#### Physical Functioning

##### Motor Symptoms of PD

**Fig. 4:**
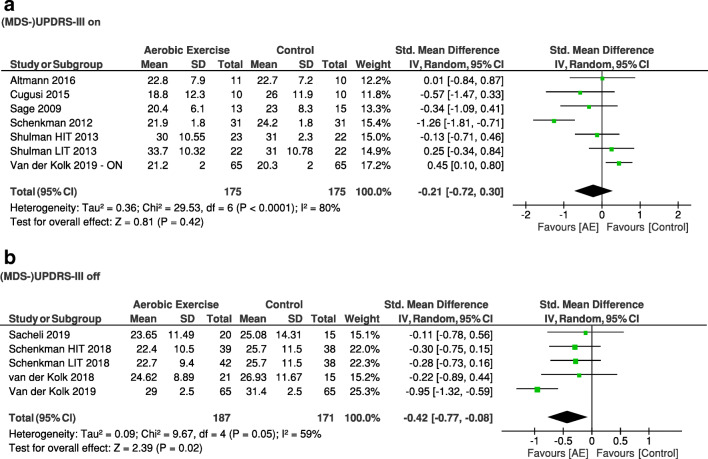
Meta-analysis on the effect of aerobic exercise on motor function in PD comparing post-intervention (MDS-)UPDRS motor section between the aerobic exercise group and the control group in the medication on (**a**) and off (**b**) state. AE = aerobic exercise group; HIT = high-intensity group; LIT = low-intensity group.

Aerobic exercise likely has a beneficial effect on PD motor symptoms (Fig. 4). Eleven randomized controlled trials investigated the effect of aerobic exercise on motor symptoms, as assessed with the (MDS-)UPDRS motor section [4, 11, 23–25, 27–31, 33]. This was the primary outcome in four studies [4, 23, 27, 30]. Three studies showed that motor symptoms either improved (when measured in the *on*-medication state) [28] or attenuated (when measured in the *off*-medication state) [4, 23]. This was not confirmed in the eight other studies [11, 24, 25, 27, 29–31, 33]. However, these latter studies were generally of lower methodological quality. Moreover, our meta-analysis shows an overall positive effect of aerobic exercise on motor symptoms in the *off* but not in the *on* state (Box 2). The long-term effects (beyond 6 months) remain unclear.

##### Other Aspects of Physical Functioning

The preceding paragraph clarifies that exercise has a beneficial effect on a composite score for multiple motor domains. Other research examined whether exercise might also benefit specific isolated motor features such as gait, balance, falls, or functional mobility, but there is insufficient evidence for a beneficial effect here. Specifically, 12 randomized controlled trials investigated the effects of aerobic exercise on these motor symptoms, with conflicting results [4, 11, 23, 24, 27–33, 35]. Two studies showed that aerobic exercise improved balance more than usual care [28, 32]. However, the improvement did not transfer to other balance tests [32] and this finding was not confirmed by other studies [4, 24, 27, 33, 35]. Because of the specificity of training, we expected that gait-based interventions, for example using a treadmill, would have more positive effects on functional mobility than non-gait-based interventions (i.e., cycling). However, this could not be confirmed by the available data, which showed that both types of interventions had no positive effect on physical functioning.

#### Non-motor Symptoms

##### Mood Disorders and Apathy

Nine studies evaluated the impact of aerobic exercise on mood disorders [4, 25, 26, 28, 29, 31–33, 36], and three studies also included apathy as outcome [25, 28, 29]. Two studies showed a positive effect of aerobic exercise on depressive symptoms [28, 32]. This was, however, not confirmed by other trials [4, 25, 26, 29, 31, 33, 36]. For apathy, only one study showed a positive effect [28]. We conclude that there is insufficient evidence for a beneficial effect of aerobic exercise on mood disorders and apathy.

##### Cognition

Six trials investigated the effect of aerobic exercise on cognition, with conflicting results [4, 25, 29, 33, 36, 37]. Two studies showed that aerobic exercise improved executive function (Stroop Test) [25, 37]. Another study found a positive effect on mental flexibility (Trail Making Test B), specifically in persons with PD with cognitive impairments [36]. The other studies showed no change in cognitive functioning measured with different instruments [4, 29, 33]. We therefore conclude that there is insufficient evidence for a beneficial effect of aerobic exercise on cognition.

##### Sleep and Fatigue

Sleep was included as a secondary outcome in three trials, none of which reported a positive effect of aerobic exercise [4, 25, 33]. Three trials also included fatigue as a secondary outcome [4, 28, 31]. One of these showed a positive effect [28] while the others showed no effect on fatigue. The study that did find a positive effect on fatigue also reported improvements in physical functioning, depressive symptoms, apathy, and overall burden of non-motor symptoms [28]. However, this study has several methodological shortcomings: the intervention was short (only 12 weeks), the sample was small (*N* = 20), and the control group was not blinded. Overall, there is currently insufficient evidence for a beneficial effect of aerobic exercise on sleep and fatigue.

##### Constipation

Constipation is a common and vexing issue for people with PD. In daily practice, physical activity is commonly recommended as one possible intervention. Only one recent study took this to the test and included constipation as outcome, but the results showed no effect [4].

#### Health-Related Quality of Life

**Fig. 5:**
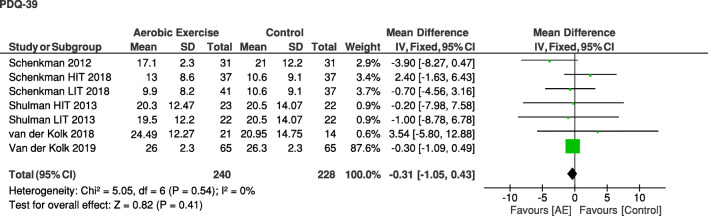
Meta-analysis on the effect of aerobic exercise on health-related quality of life in PD comparing post-intervention PDQ-39 between the aerobic exercise group and the control group. AE = aerobic exercise group; HIT = high-intensity group; LIT = low-intensity group.

Aerobic exercise should ideally not just ameliorate isolated symptoms but also improve health-related quality of life (Fig. 5). A 2-year prospective cohort study in 3408 PD patients showed that health-related quality of life decreased less in people who exercised regularly (≥ 2.5 h/week) as compared with non-exercisers [56]. However, these promising findings from observational data have not been confirmed in controlled clinical trials (which is also supported by our meta-analysis; Box 2). Only one study showed a beneficial effect of a high-intensity (80% HRmax) and high-frequency (5 times/week) aerobic exercise program [32]. The other five studies that evaluated health-related quality of life found no positive effects [4, 23, 24, 31, 33].

### Implementation Issues

#### Adherence

Although the evidence for the beneficial effects of exercise is accumulating, many patients with PD still do not adopt an active lifestyle in daily life. Many comply very well with prescribed exercise regimens within the context of clinical trials. Attrition across the aerobic exercise studies included in the systematic review ranged between 0% in small supervised trials [11, 28, 34, 36] and 25% in a program that was also personally supervised [25]. Adherence to the prescribed frequency, intensity, and duration of exercise is not often well reported. Attendance rates to on-site training sessions varied between 87 and 100% [25, 26, 28, 30, 32, 36], and adherence to exercise frequency in partly or unsupervised settings was still good (> 75% sessions attended) [4, 23, 33]. Although adherence is overall good in clinical trials, in daily life, many practical barriers must be overcome [57–64]. Barriers include a range of factors like low self-efficacy, generic health issues, poor access to the exercise location, mobility problems such as postural instability, and non-motor symptoms such as fatigue and depression [57–63, 65–71]. On the other hand, social support by family, friends, or a professional and education about the benefits of exercise can motivate PD patients to become more physically active [57, 59, 60, 62–69, 72–74]. At least 150 min of moderate-intensity exercise spread over multiple days is advised [5], but we find it helpful to instruct patients to exercise on a daily basis so it becomes part of their everyday routine. Healthcare professionals should be aware of these various barriers and facilitators, so these can be addressed and alleviated in a personalized approach, preferably using a sports coach. We have a positive experience with such coaching programs in two studies. The first was the ParkFit trial which deployed coaches who codesigned an individualized exercise program together with patients, after which patients signed a contract to commit themselves to the planned activities; compliance proved to be good [64]. The second was the Park-in-Shape trial in which coaches remotely supervised patients: they offered support, tracked progression, and adjusted the training program via phone calls; compliance was excellent [4]. Interestingly, recent advances in mobile technology promise to offer a scalable alternative for intensively supervised interventions [74, 75]. Apps could offer remote monitoring, give feedback, and allow for interaction with peers or others in the social environment. Few studies have thus far implemented such an approach in PD [4, 74], but mobile technologies certainly show promise for future exercise programs to boost compliance in daily life and may simultaneously be used to monitor their long-term effects in both research and clinical practice.

#### Safety

Although aerobic exercise studies in PD report low rates of exercise-related adverse events (five studies reported no adverse events [11, 28, 29, 32, 33], and one study reported that 2% of people [4] and another study 20.9% of people [23] in a high-intensity exercise group experienced an adverse event with a severity greater than mild, while this was the case for 8.9% of people in the moderate-intensity exercise group [23], there are some risks, especially in people with more advanced stages of PD. Obvious risks to consider are fall-related injuries and cardiovascular complications. Although the percentage of people with cardiac events that interfere with exercise was low in clinical trials, there is a theoretical concern that elderly and sedentary persons who start with a vigorous exercise program may experience cardiovascular complications. While the risk of cardiac adverse events is presumably low, the potential impact of a cardiac event is obviously substantial, including myocardial infarction or sudden death. Factors that increase fall-related injuries should be minimized by ascertaining that the exercise type matches the patient’s physical capabilities. For example, people who experience freezing of gait should prefer exercising on a stationary home trainer over a treadmill. Also, exercising during a medication *on* state and at a time of the day when the patient feels subjectively best are general rules to reduce adverse events. We tell patients to consider taking an extra dose of medication prior to their exercise, in order to ascertain a good *on *state throughout the period of exercise. It is also important to inform patients about chronotropic incompetence, which is a common sign of autonomic dysfunction in PD [76] and which makes it more difficult for some patients to achieve the same exercise-related increase in heart rate [77]. This means that standardized heart rate zone estimates based on a person’s age are often not reliable and that a (sub)maximal exercise test before commencing aerobic exercise is needed. A simple self-management advice is to dose the exercise not based on heart rate, but rather to aim for an exercise that makes the patient pant, while still being able to maintain a conversation. Another strategy is to use the BORG scale of perceived effort [78] although the patient needs to be educated in how to use this scale.

Another adverse effect to consider is that persons with PD have an increased risk of exercise-induced hypotension [79] and possibly also of post-exercise orthostatic hypotension [80]. A typical and worrisome manifestation is fainting after the patient has reached the top of a staircase, which can lead to injurious syncopal falls. Patients should be warned about this. Whether cardiovascular disease is indeed more prevalent in PD is still a matter of some debate, but like any person with known cardiovascular disease or with cardiovascular risk factors, it is prudent to consult a medical specialist before engaging in aerobic exercise. Also, some form of supervision is desirable to increase both adherence and safety. Especially heart rate monitoring allows for direct feedback to the patient and supervisor about each session’s performance and about the overall training progress, bearing the limitations of a possible chronotropic incompetence in mind. It also reveals whether the training was performed at the right intensity and might suggest the presence of arrhythmias.

## Discussion

We conclude that aerobic exercise offers generic health benefits, improves physical fitness, and offers symptomatic relief (reduced motor symptoms) in people with PD. This conclusion is supported by our meta-analyses on VO_2_max and the (MDS-)UPDRS motor section in the off-medication state. More research is needed into the effect of aerobic exercise on non-motor symptoms and health-related quality of life.

What is the clinical significance of the observed increase in physical fitness and attenuation of motor symptoms for patients with PD? First, greater physical fitness may translate into cardiovascular health benefits. In the general population, higher doses of activity are associated with fewer cardiovascular complications [38] and we expect this to be the same for people with PD. A specific benefit would be a prophylactic effect on cerebrovascular disease, which commonly appears as a comorbid condition in PD; secondary prevention could help to arrest progression of symptoms such as gait disability or cognitive decline. This assumption should now be formally studied in people with PD. Second, it remains unknown whether the effects of medication and aerobic exercise are mediated through the same mechanism of enhanced dopaminergic signaling, or whether aerobic exercise has differential and synergistic effects. The attenuation of motor symptoms measured with the MDS-UPDRS motor section was predominantly observed during the medication *off* state in two trials [4, 23], so this may help patients with response fluctuations while they wait for their pharmacological treatment to kick in again. One of these two trials found no symptomatic relief in the *on* state [4]. Most other trials assessed motor symptoms only in the *on* state and showed conflicting results [24, 25, 28, 30, 31]. One possible explanation for this absent effect in the *on* state could be that the effect of medication is simply larger than the effect of aerobic exercise. Future studies should further address this issue, by including comprehensive assessments during both the medication *off* and *on* states as well as by using longer follow-up periods. Moreover, whether exercise can reverse or attenuate the underlying neurodegenerative processes in patients with PD is still unclear. Such effects are conceivable, realizing that exercise potentially creates optimal circumstances for neuroplasticity (i.e., increased angiogenesis, improved mitochondrial function, increased neurotrophic factors) [6–8, 81–85]. Some earlier studies have certainly hinted at the possibility that (high-intensity) exercise could somehow alter the course of PD. For example, better physical fitness is associated with a larger brain volume, better white matter integrity, fewer white matter hyperintensities, and better connectivity patterns in healthy elderly persons [83–86]. Moreover, aerobic exercise afforded symptomatic improvements in neurotoxic animal models of PD, which upon postmortem brain examination proved to be accompanied by adaptive neuroplasticity that promoted an increase in dopaminergic neurotransmission [6–8, 81–85]. In persons with PD, preliminary data from small aerobic exercise studies provided similar results, suggesting enhanced dopaminergic signaling [10, 86] as well as increased brain activity in the basal ganglia and motor cortex [87, 88]. Disentangling symptomatic from possible disease-modifying effects in humans remains incredibly difficult, but future exercise trials could consider including a washout period or a delayed start design, as well as inclusion of surrogate imaging outcomes (dopamine scans and/or structural/functional MRI). This exciting area definitely warrants further research attention.

There is insufficient evidence for a beneficial effect of aerobic exercise on gait, balance, falls, and functional mobility. It is possible that aerobic exercise alone, given its relatively weak effects compared to medication that we alluded to earlier [4], is unable to overcome such rather gross deficits in axial mobility. One option is therefore to consider combining aerobic exercise with task-specific (gait) training if patients manifest impairments in gait, balance, or functional mobility.

The effects of aerobic exercise on non-motor symptoms and health-related quality of life in PD were thus far unconvincing. However, several important limitations in the available evidence must be considered here. First, non-motor symptoms and health-related quality of life were never included as primary endpoint. Five studies included improvement of non-motor symptoms as the main aim [25, 26, 28, 36, 37], but none specified one primary outcome or reported a sample size calculation (except [36]). Therefore, these studies may have been underpowered to detect improvements in non-motor symptoms or health-related quality of life. Second, study candidates were not selected based on the presence of non-motor symptoms or poor health-related quality of life. Consequently, participants manifested few depressive symptoms and cognitive deficits, while baseline health-related quality of life was high, which limited the likelihood of finding an effect on these symptoms. This is supported by a study showing that aerobic exercise did improve cognitive functioning (as measured with the Trail Making Test B) in people with PD who had cognitive impairment, as compared with usual care controls who also had cognitive impairment [37]. Third, trials were relatively short (4–24 weeks), but more prolonged interventions may be needed to find an effect. Fourth, virtually no studies reported the effect of aerobic exercise on sleep [4, 25, 33], fatigue [4, 28, 31], and constipation [4], even though all these domains are theoretically attractive targets for improvement with exercise. This area therefore warrants further research. Future trials should specifically target people with PD who experience depression or anxiety, apathy, cognitive problems, sleep problems, fatigue, or reduced health-related quality of life to elucidate the effect of aerobic exercise on these non-motor symptoms and on health-related quality of life.

While evidence for a beneficial effect of aerobic exercise is emerging, much work remains to be done. First, large trials with longer follow-up periods are needed to determine the long-term effects and compliance. Second, future studies must determine the optimal and minimal dose (frequency, duration, and intensity) of exercise. So far, only one high-quality study investigated the effect of different doses of aerobic exercise in PD [23]. To optimize the aerobic exercise training sessions, we need more studies investigating the safety and effectiveness of different doses of exercise. Third, we require trials that investigate the effect of different modes of aerobic exercise. We here reviewed studies that investigated the effect of different modes of aerobic exercise—mainly treadmill training and cycle ergometry—but none have directly compared both types. Fourth, research into the potential disease-modifying effect of exercise should be high on the research agenda. Gaining knowledge into the underlying mechanism of disease modification would also be a major motivator to get people engaged in exercise. Fifth, we need a more objective measure of PD motor symptoms. The (MDS-)UPDRS motor section is the gold standard, but the clinical relevance can be questioned as it only provides a snapshot of in-clinic performance that may not reflect actual daily life performance [89]. Wearable sensors allow for continuous and unobtrusive measurement in daily life. However, digital biomarkers derived from such sensors remain to be developed and have yet to prove their value as outcomes in clinical trials. Finally, to promote aerobic exercise even in remote, loosely populated or underdeveloped areas of the world, we need scalable approaches. Wearable sensors and remote monitoring through apps could be useful here, but their merits also remain to be proven in future trials [89].

## Electronic supplementary material

ESM 1 (PDF 508 kb)

### Electronic supplementary material

Below is the link to the electronic supplementary material.

ESM 2 (DOCX 27.8 kb)
