# Correction to: Current Perspectives on Aerobic Exercise in People with Parkinson’s Disease

**DOI:** 10.1007/s13311-022-01219-6

**Published:** 2022-03-15

**Authors:** Sabine Schootemeijer, Nicolien M. van der Kolk, Bastiaan R. Bloem, Nienke M. de Vries

**Affiliations:** Donders Institute for Brain, Cognition and Behavior, Department of Neurology, Center of Expertise for Parkinson & Movement Disorders, Radboud University Medical Center, PO Box 9101, 6500 HB Nijmegen, Netherlands

## Correction to: Neurotherapeutics https://doi.org/10.1007/s13311-020–00904-8

This Correction is to update the published article Schootemeijer et al. [1] and address concerns raised in meta-analyses.

We would like to correct minor errors in the meta-analyses on cardiorespiratory fitness, motor symptoms and quality of life in people with Parkinson’s disease (PD) and state that these corrections have a minor impact on our conclusions.

In some analyses, we erroneously used standard errors instead of standard deviations. In addition, multiple interventions from the same studies were used separately while comparing to the same control group, thereby increasing the weight of the control group. We have now pooled the interventional groups reported in Schenkman et al. [2] and Shulman et al. [3] and calculated the adjusted mean, standard deviation and sample sizes according to Higgins et al. [4]. These changes impacted the results of the previous analyses, which have now been corrected (see replacement Figs. 2–4).

**Fig. 2:**
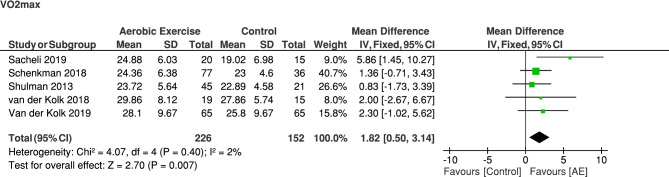
Corrected meta-analysis on the effect of aerobic exercise on physical fitness in PD comparing follow-up VO2max between the aerobic exercise and the control group. AE: aerobic exercise group

The positive effect of aerobic exercise on physical fitness is still supported by the corrected meta-analysis: the aerobic exercise group showed a higher physical fitness during a maximal graded exercise test (VO2max) at the post-assessment compared to the non-exercise or resistance exercise control groups (mean difference (MD) [95% CI]: 1.82 [0.50; 3.14], Z = 2.70, p = 0.007) (Fig. 2).

The effect of aerobic exercise on PD motor symptoms ((MDS)-UPDRS) in the off medication state is no longer significant in the adjusted analysis at post-assessment compared to the non-aerobic control group (MD [95% CI]: -0.19 [-0.42; 0.03], Z = 1.66, p = 0.10) (Fig. 3B). Although the overall effect was not significant, all studies reported a beneficial effect. The results that we now present are in line with a recent review on this topic [5]. Other corrections did not affect the conclusions. No significant difference was observed between the aerobic exercise group and a non-aerobic control group for PD motor symptoms in the medication ON state (MD [95% CI]: -0.07 [-0.29; 0.15], Z = 0.63, non-significant) (Fig. 3A) and for health-related quality of life (MD [95% CI]: -0.10 [-2.56; 2.36], Z = 0.08, non-significant) (Fig. 4).

**Fig. 3:**
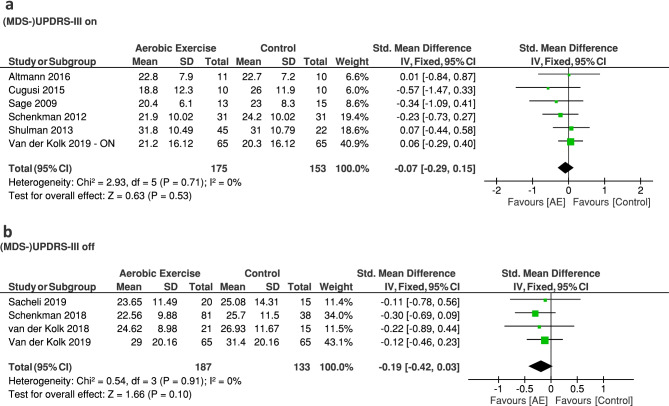
Corrected meta-analyses on the effect of aerobic exercise on motor function in PD comparing follow-up (MDS-)UPDRS motor section between the aerobic exercise and the control group. AE: aerobic exercise group

**Fig. 4:**
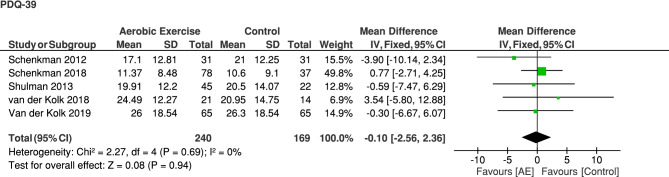
Corrected meta-analysis on the effect of aerobic exercise on health-related quality of life in PD comparing follow-up PDQ-39 between the aerobic exercise and the control group. AE: aerobic exercise group

We conclude that aerobic exercise offers generic health benefits, improves physical fitness, and shows a trend towards a positive effect on motor symptoms in people with PD. More research is needed to definitively establish the effect of aerobic exercise on motor- and non-motor symptoms and health-related quality of life.
