# Supplementary material for: Current Perspectives on Aerobic Exercise in People with Parkinson’s Disease
Source: Neurotherapeutics. 2020 Aug 17;17(4):1418–33. doi: 10.1007/s13311-020-00904-8 (PMC7851311; doi:10.1007/s13311-020-00904-8)
Supplement: Supplementary file 2 — (DOCX 27.8 kb) [file 13311_2020_904_MOESM2_ESM.docx]

**Supplementary Box 1**

**Search strategy systematic review in PubMed**

The following search string was entered in PubMed:

((Parkinson Disease [MeSH] OR Parkinson* [tiab])) AND (Exercise [MeSH] OR Exercise Therapy [MeSH] OR Endurance Training [MeSH] OR Walking [MeSH] OR Bicycling [MeSH] OR Exercise* [tiab] OR Endurance* [tiab] OR Walking [tiab] OR Bicycling [tiab] OR Ergometry [tiab] OR Treadmill* [tiab] OR Aerobic exercise* [tiab] OR Aerobic activit* [tiab] OR Physiotherapy [tiab] OR Physical therapy [tiab] OR Training [tiab] OR Cardiovascular training [tiab] OR Physical Activit* [tiab] OR Physical Exercise* [tiab] OR Rehabilitation Exercise* [tiab]) AND (Clinical Trial[ptyp] AND ( "2014/08/01"[PDat] : "2020/02/19"[PDat] ) )
